# Experimental Infection Models and Their Usefulness for White Spot Syndrome Virus (WSSV) Research in Shrimp

**DOI:** 10.3390/v16050813

**Published:** 2024-05-20

**Authors:** Natasja Cox, Evelien De Swaef, Mathias Corteel, Wim Van Den Broeck, Peter Bossier, Hans J. Nauwynck, João J. Dantas-Lima

**Affiliations:** 1 IMAQUA, 9080 Lochristi, Belgium; evelien.swaef@imaqua.eu (E.D.S.); mathias.corteel@imaqua.eu (M.C.); joao.lima@imaqua.eu (J.J.D.-L.); 2 Laboratory of Virology, Department of Translational Physiology, Infectiology and Public Health, Faculty of Veterinary Medicine, Ghent University, 9820 Merelbeke, Belgium; hans.nauwynck@ugent.be; 3 Department of Morphology, Medical Imaging, Orthopedics, Physiotherapy and Nutrition, Faculty of Veterinary Medicine, Ghent University, 9820 Merelbeke, Belgium; wim.vandenbroeck@ugent.be; 4 Laboratory of Aquaculture & Artemia Reference Center, Department of Animal Sciences and Aquatic Ecology, Faculty of Bioscience Engineering, Ghent University, 9000 Ghent, Belgium; peter.bossier@ugent.be

**Keywords:** white spot syndrome virus, infection models, virulence, pathogenesis, transmission, epidemiology, antivirals, immunomodulators, genetic selection

## Abstract

White spot syndrome virus (WSSV) is marked as one of the most economically devastating pathogens in shrimp aquaculture worldwide. Infection of cultured shrimp can lead to mass mortality (up to 100%). Although progress has been made, our understanding of WSSV’s infection process and the virus–host–environment interaction is far from complete. This in turn hinders the development of effective mitigation strategies against WSSV. Infection models occupy a crucial first step in the research flow that tries to elucidate the infectious disease process to develop new antiviral treatments. Moreover, since the establishment of continuous shrimp cell lines is a work in progress, the development and use of standardized *in vivo* infection models that reflect the host–pathogen interaction in shrimp is a necessity. This review critically examines key aspects of *in vivo* WSSV infection model development that are often overlooked, such as standardization, (post)larval quality, inoculum type and choice of inoculation procedure, housing conditions, and shrimp welfare considerations. Furthermore, the usefulness of experimental infection models for different lines of WSSV research will be discussed with the aim to aid researchers when choosing a suitable model for their research needs.

## 1. Introduction

White spot syndrome virus (WSSV) is known to be highly contagious for most of the commercially important species of penaeid shrimp and capable of causing a mass mortality up to 100% within 3 to 10 days on shrimp farms [1,2,3,4,5,6,7,8,9]. Although considerable advances have been made, such as the discovery of the nephrocomplex and its role during WSSV entry [10,11], it is generally accepted that a better understanding of the pathogenesis of WSSV is necessary to develop better control measures. This relies heavily on the development of standardized experimental *in vivo* infection models, since the establishment of continuous cell lines to aid in the investigation of threats such as WSSV is still a work in progress [12,13]. Studies have attempted to identify promising donor organs, cell culture conditions, and an appropriate medium [12,14,15,16,17,18,19,20,21,22,23,24,25,26,27,28,29,30,31,32,33,34,35,36,37,38,39,40]. Recently, a patent was filed for an improved medium exclusively for *in vitro* growth of crustacean cells [41,42]. However, the inhibition of neoplastic transformations in decapods has possibly interfered with the creation of continuous shrimp cell lines so far [13,43,44,45,46,47]. Indeed, *in vivo* infection models continue to be an important and useful tool for identifying and studying the pathogenicity of WSSV and for evaluating the efficacy of mitigation strategies to prevent WSSV outbreaks [48,49,50,51,52]. Fundamentally, a WSSV challenge procedure requires the following: (1) the use of animals with low genetic variability and high susceptibility to the virus, free of specific pathogens, (2) a WSSV stock with a known titer of infection, and (3) controlled environmental factors, such as salinity and temperature [51,53]. First, this review will critically discuss the complexities of some of the key aspects of WSSV infection model development that are often neglected and that can greatly affect the experimental outcomes. Second, this review aims to discuss the usefulness of experimental infection models for researchers in diverse lines of WSSV research. The selection of the most appropriate WSSV infection model is typically not straightforward and should be based on the research question(s) being asked [54].

## 2. Key Factors in *In Vivo* WSSV Infection Model Development

When developing a WSSV infection model, the first requirement is that the results produced by the model should be reproducible for it to be useful. Additionally, it is paramount that WSSV researchers also consider to what degree a model reflects reality to judge the relevance of its use [55]. Standardization, experimental animal quality, choice of inoculum, inoculation procedure, and housing conditions are some of the key aspects of WSSV infection model development. Unfortunately, the impact of these aspects on a model’s reproducibility and relevance is often not adequately considered [56]. This section aims to explain the complexity of each of these aspects to emphasize their influence and importance to investigators in the field of WSSV research.

### 2.1. Standardization of Disease Triad Components and Transparent Reporting

The infectious disease triad (syn epidemiological triad) describes that disease outbreaks are the consequence of exposure of a susceptible host to a virulent agent, under permissive environmental conditions. Hence, when developing *in vivo* infection models, standardization of the components of the disease triad is crucial, because each component may influence the results of the experiments that are performed [57,58]. Moreover, factors pertaining to the standardization of the disease triad should be reported to provide sufficient external validity and background for integration of the research findings within the context of the broader system [56]. These factors are reviewed by Arbon et al. [56] and adapted in Table 1. The authors gathered data from 186 peer-reviewed publications of viral challenge experiments involving Penaeus monodon. They noted that the apparent absence of reported viral (inoculum) data was particularly concerning. Most of the studies did not report the source of the study virus (71%, 132/186), with only 29% (54/186) directly reporting the geographic or genetic source, and 15% (28/186) reporting the temporal origin of the virus. This is problematic, because the lack of reported detail severely limits the potential for research to make progressive advancements [56]. A standardized and transparent infection model procedure is essential (1) to compare the susceptibility of different host species and life stages to WSSV, (2) to determine the virulence of different WSSV strains, (3) to study host–pathogen–environment interactions, and (4) to test the efficacy of strategies aimed to control the disease [51,53].

### 2.2. Experimental Animal Quality

The characteristics of the experimental shrimp, including species, life stage, genetic background, geographical source, and pre-existing pathogen infections, can potentially influence the dynamics of disease expression [56,59,60,61]. It is therefore important that these characteristics are standardized and transparently reported, as mentioned previously. This will be further discussed in Section 3.1.2. However, a shrimp characteristic that is very rarely addressed, specifically in relation to WSSV infection studies, is the “shrimp seed quality”. The term “seed quality” or “(post)larval quality” in shrimp aquaculture generally refers to the physiological condition of shrimp seed during larviculture and has a direct relationship with the survival, growth performance, resistance to stress (e.g., manipulation stress and changes in environmental conditions), and the resistance to pathogens [62,63,64,65]. The seed quality from nauplius to postlarva can be affected by broodstock condition or maternal effects, environmental and management conditions during larviculture, as well as larval diets not fulfilling nutritional requirements [62,66]. Hence, this quality can vary significantly between shrimp batches, regardless of genetic or geographic origin, and this could in turn cause variation between study results [62,63,67,68,69], e.g., the use of low-quality batches during a WSSV challenge experiment could hypothetically lead to a lower survival rate compared to the use of high-quality batches. This should certainly be taken into consideration when conducting WSSV challenge experiments and analyzing the outcomes. Researchers are advised to perform a standard quality assessment of the experimental animals (e.g., with a salinity stress test as reviewed by [70]) prior to the main infection experiment, or they could consider replicating the experiment with different shrimp batches to account for the potential variation in quality.

### 2.3. Choice of Viral Inoculum and Inoculation Procedure

The viral inoculum used for *in vivo* experimental infection studies is usually made from WSSV-infected shrimp tissues. These shrimp tissues should be free of other specific pathogens (certified specific pathogen free “SPF” status) to prevent the occurrence of co-infections that might influence the outcome of the experiments. For instance, previous studies have shown that co-infection with infectious hypodermal and hematopoietic necrosis virus (IHHNV) led to reduced infection and mortality during experimental WSSV infections in *Litopenaeus stylirostris* and *L. vannamei* [71,72,73,74]. Additionally, prior infection with WSSV reportedly enhanced the multiplication and disease-inducing capacity of *Vibrio campbellii* in juvenile *L. vannamei* [75].

The inoculum can be prepared in a liquid or a solid state of matter. Liquid viral stocks are typically processed by mincing and homogenizing infected tissues, suspending them in a buffer solution, centrifugating the suspension, and filtering or purifying it. The supernatant is subsequently collected and stored at −80 °C [51,53,76,77,78]. Apart from infected tissues, infected hemolymph has also been used as a source to produce liquid viral stock [78,79,80]. As reported by Dantas-Lima et al. [78], using infected hemolymph resulted in a stock of superior purity compared to a stock made from infected tissues after purification by iodixanol density gradient centrifugation, though the infectivity was lower. Naturally, the virus source for the liquid stock can be chosen based on a study’s requirement, but the inclusion of a purification step during processing is always recommended [81,82,83,84,85,86,87]. After all, purification of the inoculum minimizes potential confounding factors associated with less purified viral inocula and facilitates reproducible infection experiments with high accuracy [56,78].

Solid inoculum is a cruder type of inoculum that can be made from unprocessed or minced WSSV-infected tissues. Several authors have been critical about the use of this type of tissue inoculum [56,76,88], because the distribution of WSSV in the body of a shrimp is likely uneven [56,89,90]. Some studies have therefore included a mixing or blending step in their solid inoculum processing procedure to further homogenize the tissue inoculum with the aim to obtain a more even distribution of the virus [91,92]. Purification of solid tissue inoculum has, to our knowledge, not been described. The use of homogenized tissue is preferable compared to the use of less-processed viral inocula whenever possible, since it minimizes the variation of WSSV distribution in the inoculum, which could in turn positively affect the reproducibility of the results.

The choice of viral inoculum type is of course closely linked to the chosen inoculation method. Experimental WSSV infections can be induced in various shrimp species through intramuscular injection, oral intubation, immersion, co-habitation, *per os* feeding of infected tissues, or via the antennal gland. The advantages and limitations of these inoculation procedures are listed in Table 2. Intramuscular injection of the virus is typically the most effective inoculation method, and it ensures that the virus enters all animals at a predetermined dose, which is for instance a must during *in vivo* titrations of viral strains [51,53,76,93]. Still, intramuscular injection is an artificial infection route because viral particles are not confronted with the natural defense barriers of the host [94,95]. Oral intubation has been tested to make it possible to deliver a fixed quantity of virus to all inoculated shrimp via the peroral route, since this has been considered one of the natural transmission routes [4,76,96,97]. However, this is a method that requires skillfulness, and the correct selection of the inoculation device is paramount [76]. Escobedo-Bonilla et al. [98] remarked that they could not exclude that fissures were made in the cuticle of the foregut, resulting in free access of the virus to epithelial cells. Moreover, an additional disadvantage of this method is the risk that shrimp might regurgitate the inoculum [98], which invalidates the benefit of delivering a fixed dose. The intrabladder inoculation method has been recently developed as an alternative process to deliver a fixed dose via a potentially natural infection route [99]. Hereby, a catheter is inserted in the terminal duct of the ventral urinary bladder to inject a liquid [10]. This technique has been described to be very effective, though it might also require personnel that are well-experienced in performing the procedure [10,58,100]. Experimental infections through immersion, co-habitation, or feeding are less invasive methods, which might also represent natural WSSV transmission routes [88,93]. Transmission through rearing water has often been viewed as a less effective path of infection compared to consumption of infected tissue, by cannibalism or predation [4,96,97]. However, this idea has been contested by Tuyen et al. [101], who found that indirect water-borne transmission was more important than direct transmission through cannibalism in *L. vannamei*. A more recent study attempted to explain these contradictive findings, postulating that cannibalism can facilitate direct peroral WSSV transmission by ingestion of infected tissues, or it can promote indirect water-borne WSSV transmission because the act of chewing potentially releases multiple virus particles in the water, immersing the cannibal and other shrimp in its vicinity [102]. This suggests, however, that an *in vivo* infection model that mimics or leaves room for the occurrence of cannibalism might provide better insights into the natural infection dynamics [93]. However, it also risks becoming less controllable as discussed in the next section, Section 2.4.

### 2.4. Housing Conditions

Choosing the housing conditions for a WSSV infection model is extremely important albeit complex, because it determines, for the large part, which information a researcher will be able to obtain and possibly also the validity of that information. If animals are housed in a group, for instance, it can often not be guaranteed that every animal receives the same amount of infectious virus during a challenge with WSSV-infected tissues [76,88,113]. Individual housing of the experimental animals offers a potential solution to this issue [76,102,113]. Indeed, a WSSV challenge model in which shrimp are individually housed has certain advantages over a WSSV group challenge model. It allows for the collection of research data in a more controlled scientific setting. The amount of infectious virus consumed by each shrimp can be monitored and recorded. This is especially useful during *per os* feeding of infected tissues, because in this case, it is unlikely that all individually housed animals eat the same amount of tissue at the same time [113]. In addition, the clinical outcomes on the level of the individual shrimp can be evaluated [102,123]. On the other hand, a challenge in a group simulates more closely the on-farm reality of a WSSV outbreak, as it allows for disease transmission between shrimp [102]. This might indeed generate results that can potentially be easier to extrapolate to the field, but due to the less controllable nature of this experimental setting, results may be less reproducible and accurate data collection becomes more challenging. Nevertheless, both model types can be very useful and complementary tools to use in WSSV research [102,123]. The choice for individual or group housing may also be largely determined by the specific research question that one desires to be answered [54]. For instance, individual infection models might be better suited for research on genetic parameters of resistance to WSSV and characterization of a therapeutics’ dose–response [113,121]. Conversely, the use of group infection models for therapeutic efficacy trials might benefit the extrapolation of laboratory findings to the field [123]. This will be further discussed in Section 3.

Another important issue to note is that whatever type of housing is chosen, the water temperature and salinity in the experimental units should always be controlled so that the values of these environmental parameters fall within the acceptable ranges. Especially, water temperature, a parameter with a profound effect on the severity of a WSSV outbreak, can be difficult to control in a complex and large experimental setting [124,125]. Nevertheless, it is extremely important that the water temperature in the experimental units does not fluctuate over time, but also that there are no hot or cold spots within the same experimental unit. After all, WSSV appears to be most virulent in water temperatures between 25 and 28 °C, but shrimp maintained at low (12–15 °C) or high temperatures (>32 °C) exhibited decreased/deferred death [114,124,126,127,128]. Moreover, a recent study has demonstrated that shrimp migrate to warmer areas to raise their body temperature to limit WSSV infection and increase their survival capacity. This phenomenon is called “behavioral fever” [42] and could gravely affect the outcome of a WSSV infection experiment.

### 2.5. Shrimp Welfare Considerations

Historically, ethical and regulatory oversight of research animals has been mostly focused on vertebrates and rarely included invertebrates, such as shrimp. After all, it has not yet been conclusively demonstrated that decapods possess sensory structures of sufficient complexity to feel pain [129,130,131]. Nevertheless, there has been a growing awareness amongst researchers, producers, policy makers, and other stakeholders that the lack of conclusive evidence on the sentience of penaeid shrimp, should not be confused with the absence of sentience in these organisms [129,130,131,132,133,134,135,136]. Indeed, as reviewed by Pedrazzani et al. [129], several countries in Europe have started to include crustaceans in animal welfare legislation [129,130,137,138,139,140], although experiments in shrimp—as they are invertebrates—are still exempt of ethical approval as described in Directive 2010/63/EU of the European Parliament and of the Council of 22 September 2010 on the protection of animals used for scientific purposes. In Asia, where some of the world’s largest shrimp producers are located, codes of conduct already include animal welfare as one of the pillars, along with food safety, animal health, and environmental integrity [129,141]. Thus far, the United States has prioritized economic concerns in conflicts between trade and animal welfare [142].

The necessity for rigorous oversight is being recognized, but it is important that the associated bureaucracy is not allowed to become prohibitive, causing scientists to avoid pursuing justifiable and important research involving animals [133]. Public support for research is conditional—animals should not suffer unnecessarily, and sufficient potential benefit should accrue from the research. However, society also actively seeks pioneering medical and scientific advances which can only be achieved through research [133,143]. Animal models and cell culture systems are essential for scientific advancement in WSSV research, to understand the complex interaction between the host and pathogen in the aquatic environment and to enable the development of safe and effective vaccines and therapeutics against WSSV [51,53,54,88,97,98,101,102,115,120,144,145,146]. Hence, the choice of model must be a thoughtful and clearly defined process considering the model’s advantages and limitations. Moreover, experimental infection models must be well characterized and understood to avoid making erroneous conclusions, hindering scientific advancement and resulting in a waste of animal life [54].

## 3. Usefulness of *In Vivo* WSSV Infection Models Based on Research Needs

The statistician George Box brought forward the famous quote, “All models are wrong, but some are useful”. This implies that a model’s usefulness is related to its concrete purposes and applications [55]. Indeed, although reproducibility and relevance are important as mentioned in the introduction of Section 2, the true value of a WSSV infection model can only be determined in the context of a precisely defined research question and even though no model is 100% correct, its use can be justified based on the research needs [55]. The following sections will further elaborate on this matter with the aim to provide investigators with solid arguments for or against the selection of certain types of WSSV infection models in specific research contexts.

### 3.1. Evaluation of WSSV Virulence

In invertebrate pathology, virulence has been defined as the greater or lesser capacity of an infectious agent to provoke disease after having infected the host. It is, in other words, the degree of pathogenicity within a group or species [147,148]. Virulence is determined by a combination of virus and host factors [149,150,151]. Understanding the role of these factors in WSSV pathogenesis requires *in vivo* studies [149].

#### 3.1.1. Virulence According to the Strain

Historically, the development of valid methods to properly evaluate the virulence of WSSV strains has been a challenge. Shortly after WSSV first appeared, several experiments were conducted to demonstrate the virulence of WSSV in crustacean hosts [152,153,154,155,156]. From these initial experiments, however, the virulence of WSSV could not be determined, because the dose of infectious virus that was given to the individual animals was unknown [53]. To overcome this issue, molecular techniques such as competitive polymerase chain reaction (PCR) to quantify viral loads in infected tissues were developed, but these techniques could not discriminate between infectious and non-infectious viral DNA [157]. In fact, the matter is even more complicated, because although the presence of viral DNA may correlate with the infectivity of a virus strain, this does in theory not automatically confirm the causal relationship with disease. Infection is, after all, the term that defines the entrance and development of an infectious agent in a human or animal body, whether it develops into a disease or not [51,147]. Indeed, researchers in the field noted that WSSV infection can be detected in shrimp sampled from ponds in which there are no indications of a disease outbreak [146,158,159]. However, a study by Ngo et al. [146] that encompassed a combination of laboratory dose–response experiments, infection modelling, and meta-analysis, estimated a large probability of host death upon WSSV infection for all its models and data sets [146]. Since the infection status of shrimp in most of these studies was determined by PCR, it was indicated that there was generally a strong correlation between the presence of WSSV DNA and disease. Nevertheless, in and of itself, the quantification of WSSV DNA does not allow for a direct evaluation of the disease producing power or virulence of a WSSV strain [51,160,161,162,163,164,165,166,167]. To compare the virulence of different virus strains, first and foremost, the ability of an infectious agent to cause a new infection in a susceptible host should be known [115,147]. To date, in the field of virology, different methods have been used to determine infectivity depending on the virus concerned. The most often used traditional methods are the plaque forming units (PFU) assay and its derivative the 50% tissue culture infective dose (TCID_50_) assay. These methods are based on serial dilutions of the virus-containing samples and observation of the appearance of a cytopathic effect (CPE) in a cell monolayer [168,169]. Quicker and less laborious titration procedures have also been developed that depend on the identification of singular infected cells using immunodetection of viral proteins by flowcytometry [168,170,171,172,173]. Unfortunately, the lack of immortal crustacean cell lines has been hampering the *in vitro* determination of WSSV infectious titers. The use of primary crustacean cell culture systems for titration of WSSV has been investigated, but these systems have not yet reached a sufficient level of reproducibility and standardization [174,175,176,177]. Consequently, standardized methods were published to determine the shrimp infectious dose 50% endpoint (SID_50_) and the lethal infectious dose 50% endpoint (LD_50_) during controlled *in vivo* titration experiments [51,53,105]. These *in vivo* titration techniques use a standardized intramuscular inoculation procedure to inject shrimp of the same age with serial dilutions of a WSSV-containing sample. These shrimp are then housed individually, to prevent potential host-to-host transmission. The virus infection titers (SID_50_ mL^−1^) and mortality (LD_50_ mL^−1^) can subsequently be calculated using the method of Reed and Muench [178]. Since this procedure was described in shrimp [51,53,105], it has been used in multiple studies to determine the infective titers of WSSV stocks so that a known dose of infectious virus could be administered to a group of susceptible individuals [10,76,102,115,121,179]. Through this method, Rahman et al. [115], for instance, managed to demonstrate that virulence differences exist between WSSV geographic isolates. This confirmed similar suppositions made in previous studies [115,118,153,165].

Although, intramuscular injection is indeed considered a more artificial inoculation method, it is still the preferred approach for *in vivo* titration of WSSV stocks in shrimp [105]. After all, oral challenge does make it more difficult to ensure that all individuals receive equal viral doses, as previously discussed [98]. It does, therefore, not allow for the accurate determination of an LD_50_ as do immersion and injection challenges [51,105,180]. Immersion on the other hand is also less recommended compared to injection inoculation since reproducible results can be harder to achieve due to water fouling issues caused by the high doses needed to obtain an LD_50_ for WSSV, as remarked by Laramore et al. [105].

#### 3.1.2. Virulence According to Host Factors

Host factors can affect the virulence phenotypes of viral strains [157,181,182]. Thus, although intramuscular injection is considered an acceptable standard method to evaluate virulence according to the viral strain (see Section 3.1.1), the virus artificially by-passes the natural physical and immune barriers of the host (see Section 2.1). Hence, a more natural infection method, such as co-habitation, immersion, or feeding of infected tissues, should be considered when a potential host’s susceptibility to WSSV is investigated. These natural infection methods are expected to reflect the experimental animal’s natural susceptibility to WSSV [93]. Following the infection experiment, further testing is necessary and can be conducted by any one or a combination of the following assays: (1) immunohistochemistry to detect infected tissue using virus-specific antibodies; (2) electron microscopy (EM) to view virions in infected cells; (3) reverse transcriptase PCR (RT-PCR) to detect viral mRNA; (4) sequencing to compare the DNA sequence to known sequences in public databases like NCBI, which allows the genetic basis of important phenotypic characteristics, such as antigenic determinants, to be elucidated, or to address more fundamental questions relating to the evolution of WSSV strains [183,184]; or (5) qPCR to show the approximate number of DNA copies/μL in a specimen—thus the potential viral DNA load [93,177].

Known risk factors that can influence susceptibility to WSSV are host species, age, physiology, and genetic background [56,157,181,182]. Standardization of these factors in a WSSV infection model will increase its reproducibility and contextualize the results, which can enhance their relevance [55,56].

##### Species-Susceptibility

WSSV’s reported host range currently includes seventy-three species of shrimps, crabs, lobsters, freshwater shrimp and crayfish, brine shrimp, copepods, and polychaetes [93,152,185,186,187,188,189,190,191,192,193,194,195,196,197,198,199], but the degree of species susceptibility to WSSV varies widely [200]. Penaeid shrimp species are highly susceptible and WSSV infection often results in high mortality. In contrast, crabs, crayfish, freshwater prawns, spiny lobsters, and clawed lobsters are also susceptible to WSSV infection, but morbidity and mortality because of infection is highly variable [201]. A study by Waikhom et al. [181] even suggested that WSSV genotypes can vary upon passage in different hosts and that differential passaging of WSSV of the same strain can cause variations in species susceptibility to that strain [202]. The authors passaged different WSSV isolates that caused 95% mortality in *P. monodon* through crabs (*Portunus sanguinolentus* and *P. pelagicus*) and shrimp (*M. rosenbergii*). These animals were confirmed to be WSSV-negative by PCR testing before the experiment. At the end of these infection experiments, the host species were frozen after confirmation of WSSV-infection, and then fed back to P. monodon to study changes in pathogenicity. Indeed, WSSV pathogenicity was altered by passaging through *P. pelagicus* but not through *P. sanguinolentus*. Additionally, passaging through *M. rosenbergii* appeared to reduce its virulence to *P. monodon*, while passage of WSSV through *P. monodon* did not result in any attenuation of the virus. Nevertheless, these observations were not in accordance with the study carried out by Pradeep et al. [203], as remarked by Shekar et al. [202]. These authors did not see a difference in virulence when passaging their WSSV isolates in shrimp (*P. monodon*) and crabs (*Scylla serrata*). However, it should be noted that Pradeep et al. [203] passaged the isolates via injection, thereby by-passing the natural defense barriers of the host. This, in combination with the use of a different species of crab, could have resulted in a different outcome.

##### Life Stage-Susceptibility

When considering decapod shrimp, all life stages are potentially susceptible to WSSV infection, although the degree of life stage susceptibility reportedly varies between species of shrimp. For instance, in *M. japonicus*, WSSV did not display pathogenicity in the larval and early post-larval stages younger than PL6 following an immersion challenge in 2 L beakers for 1 h, after which the animals were rinsed for 3 min in a net with flowing seawater. Between PL6 and PL12, however, the susceptibility to WSSV increased with the progress in development stage [204]. Virulence of WSSV in *P. monodon* was found to be less pronounced in the early life stages, but more in the later stages. WSSV challenges were performed by immersion and oral routes [59]. The early life stages of *M. rosenbergii*, on the other hand, were more vulnerable to WSSV than the later stages after oral feeding of WSSV-infected tissues [205]. In *L. vannamei*, a general tendency towards higher susceptibility associated with older ages was detected [91,95]. Pérez et al. [91] could not induce mortality before the PL30 stage in *L. vannamei* after immersion and oral challenges with WSSV-infected tissues, and the highest mortalities were obtained in PL40 shrimp, but PL stages older than PL40 were not tested. Nevertheless, these results were contradicted by a more recent study [206]. *L. vannamei* seedlings with an average weight of 4.2 mg were challenged via WSSV immersion at 10 larvae/well in 6-well plates containing 6 mL of aquacultural water and these larvae, that did not reach the PL stage yet, were confirmed to be infected through qPCR diagnostics [206].

##### Physiological Susceptibility

Studies on how the physiological condition of a shrimp (weight, molting stage, etc.) can influence its susceptibility to WSSV are a bit sparser. A recent study showed that bodyweight of post-larval *L. vannamei* shrimp had a bearing on their susceptibility to the virus. PL of the same age group and family were grouped according to bodyweight (10–20, 30–40, and 50–60 mg) and challenged through immersion. It was observed that the PLs became susceptible to WSSV at ≥50 mg bodyweight [207]. Furthermore, to investigate the susceptibility of *L. vannamei* shrimp to WSSV during the different phases of their molt cycle, shrimp in different molt stages were inoculated with WSSV either via immersion or via intramuscular injection. This study proved that shrimp are more susceptible to WSSV via immersion after molting than in the period before molting, while this difference in susceptibility was not observed after an intramuscular WSSV inoculation [95].

##### Genetic Susceptibility

Susceptibility or resistance to disease is a complex quantitative trait that is likely to be regulated by the additive effects of many genes, epigenetics and by the environment [208]. Breeders have attempted to produce WSSV-resistant shrimp stocks with varying degrees of success. Estimates of the heritability of WSSV resistance in *L. vannamei* are generally rather low, ranging between 0.01 and 0.31 depending on the batch of shrimp, the trait analyzed (e.g., days survival or binary dead or alive), the challenge method applied, and the statistical models used for genetic parameter estimation [113,209,210,211,212,213]. Moreover, limited evidence has been found for genetic variation in resistance to WSSV in *P. monodon* [214]. However, it was suggested by some authors that the lack of reported quantitative trait loci associated with WSSV resistance may not be due to the lack of segregating genes for resistance but could instead be due to the highly virulent nature of WSSV, challenge testing methods that do not deliver accurate resistant phenotypes, and because marker resources do not sufficiently cover the genome [208,209,210,211,213]. Indeed, Lillehammer et al. [213] reported that significant useful genetic improvement for WSSV resistance can be achieved in a breeding program for *L. vannamei* by applying genomic selection. With regards to the choice of an appropriate infection model that can be used for genetic selection, refer to Section 3.4.3.

### 3.2. Pathogenesis Studies

There is still an ongoing debate about the true site(s) of WSSV entry [215]. Clues to the portals of WSSV entry could be found by identifying the primary sites of WSSV replication. This has been done in several species of shrimp, including *L. vannamei* [98], *P. monodon* [110,216], and *M. japonicus* [89], with the primary sites of WSSV replication being identified as the epithelial cells of the foregut (digestive system), gills (respiratory system), and antennal gland/nephrocomplex (excretory system) [98]. WSSV spread to target organs is thought to happen as follows: after primary replication, newly produced WSSV is released from epithelial cells to cross the basal membrane and reaches the underlying connective tissues and associated hemal sinuses. However, the role of hemocytes in the systemic spread of WSSV is another topic of debate further complicated by the potential differences between shrimp species. Circulating hemocytes did not appear to play a large role in the systemic spread of WSSV in *L. vannamei* infected via peroral intubation or in *P. monodon* that were fed WSSV-infected tissues [98,217]. Viral replication in nuclei of non-circulating hemocytes of orally infected *P. monodon* shrimp on the other hand was confirmed [217]. Yet another study differentiated between the type of hemocytes that were infected, reporting that WSSV infected semi-granulocytes and granulocytes in *P. merguiensis* but not hyalinocytes [218]. Following the systemic spread, the gills, foregut, integument, and antennal gland were demonstrated to be the main targets for WSSV. [219,220]. Urine was found to be qPCR positive for WSSV (although the infectivity of the urine was not tested) [10], indicating that the antennal gland might serve as a portal of exit.

The preferred method to carry out studies on the pathogenesis of WSD is by performing an *in vivo* time course study of WSSV infection. Escobedo-Bonilla et al. [98], for instance, performed a time course study with *L. vannamei* shrimp. The animals during this study were inoculated with the WSSV Thai-1 strain via peroral intubation and collected at 0, 6, 12, 18, 24, 36, 48, and 60 hpi. WSSV infection was analyzed via immunohistochemistry (IHC). During this challenge, however, shrimp were housed in groups of six shrimp per 50 L aquarium. Since, a more recent study showed that host-to-host transmission of this WSSV Thai-1 strain in *L. vannamei* occurs between 30 and 48 hpi [102], it might have been advisable to house the shrimp in this time course study individually to ascertain that time zero was the actual start of the WSSV infection for every infected shrimp.

### 3.3. Studies on Transmission Dynamics and Epidemiology

Experimental studies on WSSV transmission and epidemiology have been scarce [97,101,102]. Ideally, this type of research should involve a combination of individual and group infection experiments to determine the relation between the disease progress in an individual host and the transmission in a population [221,222,223]. Since WSSV reportedly uses more than one infection route (e.g., water-borne [88,101], peroral [216,224], and transovarial [225,226]), each single aspect of the set-up must be considered very carefully to avoid drawing erroneous conclusions when analyzing the results [221,222,223]. For example, the results of a recent study by Cox et al. [102] in *L. vannamei* showed that individually housed WSSV-infected shrimp started shedding viral DNA in the water within 6 h of clinical disease onset and this shedding reached a peak around the time of death. These shrimp had been inoculated naturally via feeding on infected tissues but were transferred to WSSV-free tanks immediately following the inoculation. This ensured that WSSV DNA from the infected tissue inoculum would not contaminate the water samples taken during the experiment. A study by Kim et al. [104] in *L. vannamei* solved this issue by opting for an intramuscular inoculation, although it should be noted that the viral shedding rate might possibly be different based on the inoculation method. It could, therefore, be advisable to choose an inoculation method that mimics natural transmission more closely [88]. Additionally, Kim et al. [104] showed that shrimp started shedding WSSV DNA from one day post inoculation onwards at a shedding rate that increased over time. However, the shrimp in this study were housed in groups and in such a set-up where cannibalism is difficult to prevent. The viral shedding detected in this study can therefore not be directly compared to the viral shedding of the individually housed shrimp in the study by Cox et al. [102]. While the authors in the latter study would have detected natural viral DNA shedding by an infected host without interference of a cannibalizing co-habitant [102], the viral DNA detected in the water during the former study [104], could have been from a combination of natural shedding by sick hosts and the release of viral particles in the environment by cannibalism [102]. It is important to make this distinction because it can have consequences for the interpretation of the results. For example, studies by Soto and Lotz [98] and Tuyen et al. [101] used different set-ups for their experiments to investigate which mode of transmission is the most important. The authors of the former study carried out a co-habitation experiment (co-habitation with sick shrimp without cannibalism) and an ingestion (mimicking cannibalism) experiment in separate aquariums, concluding after comparison of both groups that transmission by ingestion was seemingly the most important mode of transmission for WSSV [97]. However, this conclusion suffered from the unconscious bias that because cannibalism involves the ingestion of infectious tissues, it is this ingestion that causes the transmission of WSSV [102]. When Tuyen et al. [101] conducted their pairwise co-habitation experiment, only pairs of shrimp were able to have direct contact with each other, but all these pairs were housed in cubicles in the same tank sharing the same rearing water. The authors concluded that indirect water-borne contact was more important for the transmission of WSSV than direct contact. However, they also considered cannibalism to only be a co-factor of direct contact transmission, and they did not associate it with indirect environmental transmission [102]. Cox et al. [102] proved that the act of cannibalism could facilitate water-borne virus transmission by dissemination of infectious WSSV particles by conducting an experiment where groups of shrimp were allowed to feed on WSSV-infected tissues (cannibalism) at the start. Subsequently, these groups were immediately removed from the tank set-up before they could start shedding the virus naturally, and naïve groups of shrimp were used to repopulate the tanks. These naïve shrimp were successfully infected by the WSSV-contaminated water as a result. Furthermore, the authors also showed that ingestion of WSSV-infected tissues did not significantly increase the mortality due to WSSV during an epidemic compared to immersion into water in which cannibalism had occurred. They concluded that direct WSSV transmission, through the ingestion of infected tissues, potentially plays a less important role in WSSV transmission than previously thought.

Finally, although the World Organization for Animal Health (WOAH) suggests that non-lethal screening by PCR can be performed by collecting hemolymph or pleopods [177], researchers should be aware of the potential impact of this procedure on the outcome of WSSV transmission or epidemiological experiments especially. After all, it was shown that removing a pleopod during an immersion challenge facilitates infection [95]. It might therefore complicate the interpretation of the results.

These examples show that the execution of an *in vivo* WSSV transmission experiment for WSSV in shrimp is often less straightforward than it seems, because it involves complex interactions between virus and host in an aquatic environment. The experimental designs should therefore be very carefully considered [221].

### 3.4. Testing Control Measures against WSSV

Although our understanding of WSSV infection and pathogenesis in shrimp is incomplete, the urgent need for effective control measures has led to the exploration of multiple potential mitigation strategies [96]. Antiviral therapies [227,228,229], the use of immunomodulators [230,231,232,233,234,235], genetic selection for WSSV resistance [210,211,213], and even artificial intelligence (AI) could play a significant role in WSSV mitigation [236,237,238,239,240,241,242,243]. The use of high throughput standardized *in vivo* challenge tests is commonly deemed to be a necessary and appropriate method to support their development [88].

#### 3.4.1. Antiviral Therapies

Once virus infection is established in a host, antiviral therapy aims to control it [244]. In principle, all the steps in the virus life cycle can be explored as molecular targets for antiviral therapy [244]. Indeed, our understanding of the replication cycle of WSSV is still rudimentary and based on hypotheses that are currently challenging to test, because stable continuous shrimp cell culture systems are not available yet [12,13,145,174]. Even so, anti-WSSV therapy development is in a state of flux which can largely be attributed to the research that is being conducted using *in vivo* infection models [96,245,246,247,248,249,250].

##### Biologically Active Compounds

Several studies have focused on screening plant extracts and phytochemicals as potential sources of anti-WSSV molecules [206,227,228,229,251,252,253,254] with some degree of success as reviewed by [96]. Additionally, silver nanoparticles (AgNPs) have shown antiviral activity against WSSV in shrimp [255,256,257]. Unfortunately, how these biologically active components interact with the host, virus, or the environment to produce their protective effects is often not well understood [227,228,229,251,252,253,254]. Possible modes of action against WSSV were proposed: (i) viral inactivation due to the interaction between the extract and the envelope protein, (ii) influence of the extract on the replication cycle of the virus, which prevents virus multiplication in a host cell, and (iii) immunostimulatory activity [254].

To determine if an anti-WSSV product candidate has virucidal properties due to its interaction with the viral envelope or interference with the viral replication cycle, it is recommended to carry out *in vivo* antiviral activity tests (Table 3). Using the term “*in vivo*” could be misleading though, since this type of testing is at least partially performed in an “*in vitro*” set-up [254,258,259]. Equal mixtures of a WSSV suspension (from a titrated viral stock) and a solution of the active compound are prepared *in vitro*. The active compound solution can be mixed with the WSSV suspension at different concentrations to assess the dose–response relationship [259]. Subsequently, these mixtures can be injected intramuscularly to evaluate the compound’s antiviral properties *in vivo*. If researchers wish to determine the active compound’s time to effectiveness, they can also opt to incubate the mixtures *in vitro* for some time prior to injecting the shrimp. Next, the survival of the injected shrimp can be monitored to determine the potency of the active compound [254]. This preliminary antiviral screening method is reliable to determine if a novel active compound possesses antiviral properties against WSSV. Moreover, multiple biologically active components can be screened in parallel, which reduces the costs and the time spent on development [254,258,259].

Once the antiviral properties of an antiviral drug candidate have been demonstrated, the active compound might be included in the formulation of medicated feeds or functional feed additives [254]. However, it is generally recommended to first test if the chosen delivery method manages to deliver a sufficient concentration of the active substance to the intended site(s) of action, especially if a novel drug delivery system (NDDS) is being used [260]. After all, if this is not investigated, the use of an inadequate delivery method could result in therapy failure due to poor bioavailability [261]. Delivery methods could be tested *in vivo* by intramuscular or peroral administration of the active compounds and further detection in different shrimp body tissues in a time course study. Once the delivery methods have been optimized, the efficacy of the therapy can be tested during *in vivo* infection experiments. For these experiments, it is advisable to use an inoculation procedure that resembles natural infection, as discussed in Section 2.3, because this is expected to help translate study results to the field [88,254,262]. However, it would be recommended to house the shrimp individually instead of in groups when the delivery methods and the efficacy of the therapy still needs to be established (Table 3). When the experimental animals are housed individually, the exact amount of medicated feed consumed by each shrimp can be recorded and potential individual therapy failure due non-adherence can also be recognized. In addition, individuals can easily be monitored and sampled, as discussed in Section 2.4. At a later development stage, once formulations and delivery methods have been optimized, it is more beneficial for the extrapolation of the results to opt for a set-up in which shrimp are housed in groups (Table 3). Though it also introduces more potential variables (cannibalism, competition for medicated feed) compared to individual housing, biotic or abiotic parameters, such as water temperature, salinity, presence of additional pathogens, management practices, and factors of host-sensitiveness to infection, such as physiological states, age, molting, or/and genetics, can still be controlled [57]. It is therefore recommended to test the efficacy of treatment candidates in these controlled conditions, before proceeding to assess their effectiveness under “real-world” conditions in field studies on farms.

##### Antibody Therapy

After immunizing animals with target antigens, egg yolk immunoglobulin (IgY) from hens as well as antibodies from mammals can be collected and used as a passive vaccine for shrimp against WSSV [263,264,265]. However, this technique is only used to transfer active, ready-made anti-WSSV antibodies, usually to very recently exposed individuals, and the protection is immediate but relatively short-lived [233,266,267]. There is no attempt to trigger any protective ability by the shrimp themselves [233]. In other words, anti-WSSV antibodies are virus-directed and act as neutralizing agents [268]. The proposed mechanisms of neutralization range from those requiring binding of a single antibody molecule to the virus envelope proteins to those requiring substantially complete antibody coating of virus [268].

To evaluate the neutralizing capacity of anti-WSSV antibodies against free virions, it is recommended to use the same methods as described in the section Biologically Active Compounds [265,268] (Table 3). However, it should be noted that evidence for several viruses indicates that lower antibody concentrations are required to inhibit infection propagated by free virus than are required to inhibit infection propagated by cell-to-cell spread *in vivo* [268,269,270,271]. It is therefore highly recommended to also question how well prior neutralization of anti-WSSV antibodies *in vitro* correlates with protective activity *in vivo* during a natural or induced WSSV infection. This can be tested by challenging shrimp with WSSV via a route that mimics natural infection, while administering the protective antibodies via intramuscular injection, immersion, or the feed shortly after or before the WSSV challenge [265,272]. Ideally investigators use a WSSV inoculation method that mimics natural infection (refer to section Biologically Active Compounds) (Table 3). Individual housing of the shrimp is advised in such a study to decrease the occurrence of noise in the data set due to cannibalism or reinfections via host-to-host transmission (Table 3). This could possibly complicate the correct interpretation of the results, as seen by Chen et al. [272]. These authors suggested that losses in the antibody-treated groups of *P. monodon* juveniles in their study were possibly caused by cannibalism and not by a treatment failure. However, they did not conduct a diagnostic test on the carcasses to verify their suspicions. In truth, this might have been difficult to do, because when shrimp cannibalize their conspecifics—this happens with a higher probability when their prey has recently molted—they might completely consume their prey or leave only a few pieces behind [273]. To conclude, once the neutralizing capacity of anti-WSSV antibodies has been assessed *in vivo* in individually housed shrimp that were challenged with WSSV, their potential as a treatment against WSSV might also be tested in a group challenge model (refer to section Biologically Active Compounds).

#### 3.4.2. Immune System Modulation

Immunomodulators are defined as interventions that target the host rather than the pathogen, modulating the immune response with the aim of disease prevention or treatment [274]. Their actions can be nonspecific or specific. Nonspecific immunomodulators are used to stimulate the immune response of shrimp without directing the activity of stimulated cells to a specific antigen [275,276]. Immunomodulation is specific when the stimulation translates into an immune reaction to a particular antigen, as in the case of RNAi-based therapy or vaccines [275,276].

##### Nonspecific Immunomodulators

To understand the mechanism of non-specific immunomodulators, more often referred to as immunostimulants in shrimp aquaculture [234,235,277,278,279], it is required to briefly explain these invertebrates’ well-developed innate immune system. Unlike vertebrates, shrimp do not have a separate lymphatic system. The main circulating fluid in their bodies is referred to as hemolymph because it functions as a combination of blood (hemo or haemo [Latin]) and the colorless fluid of the body (lymphae water or clear water [Latin]) [280]. Hemolymph comprises cells, water, and dissolved inorganic salts and proteins, among which the oxygen carrier, hemocyanin, is the most abundant. This copper-based protein turns blue in color when oxygenated, thus giving hemolymph a blue-green color rather than the red color of vertebrate blood [281,282]. The free cells found in hemolymph are accordingly named hemocytes. They are depleted during an immune response or by the normal aging of cells and are replenished from the hematopoietic tissue (HPT), which occurs as a series of ovoid lobules forming a thin sheet on the dorsal part of the foregut in decapod crustaceans [280,281,283,284,285]. Circulating hemocytes are typically divided into three different classes, based on cytoplasmic granularity, staining properties, density, and nuclear size, (i) hyaline cells (HC) (agranular), (ii) semi-granular cells (SGC), and (iii) granular cells (GC) [281,286,287,288,289,290,291,292], though five subpopulations were isolated in *L. vannamei* by employing a new iodixanol density gradient centrifugation other than the traditional procedure in Percoll [292]. The three main classes are involved in important immune functions. In general, HCs are responsible for phagocytosis, while SGCs are involved in encapsulation, melanization, and coagulation, along with phagocytosis in some species, such as *M. japonicus* [293,294] and *Macrobrachium rosenbergii* [295]. The GCs with numerous big eosinophilic granules participate in the storage and release of the prophenoloxidase (proPO) activating system, melanization, cytotoxicity, secrete antimicrobial peptides (AMPs), such as penaeidins, and interact with the envelope proteins of WSSV to block multiple viral infection processes, thereby protecting the host against WSSV [281,296,297]. In short, innate invertebrate immunity is mediated by circulating hemocytes [298,299], and hemocytes generate germ-line encoded pattern recognition receptors (PRRs) [300,301], such as peptidoglycan recognition proteins (PGRPs), Gram-negative binding proteins (GNBP) or lipopolysaccharide and b-1,3-glucan binding proteins (LGBPs), C-type lectins, galectins, thioester-containing proteins (TEPs), fibrinogen-related proteins (FREPs), scavenger receptors (SRs), Down syndrome cell adhesion molecules (Dscams), and Toll-like receptors (TLRs) [301]. PRRs recognize pathogens by binding to molecular patterns (molecular structures) rather than to a specific component of a specific pathogen. These pathogen-associated molecular patterns (PAMPs) are typically shared by a group of microbes and are essential for their survival. Known PAMPS are polysaccharides and glycoproteins on the surface of microbes, such as lipopolysaccharide (LPS) from Gram-negative bacteria, peptidoglycan (PGN) and lipoteichoic acid (LTA) from Gram-positive bacteria, and glucans from fungal cells. PAMPs can also be polynucleotides, such as bacterial and viral unmethylated CpG DNA, and single-stranded and double-stranded RNA from viruses [301,302]. The recognition process leads to rapid humoral and cellular immune responses [298,301,303].

Similar to any infection, immunostimulants also get detected as foreign material when they enter the shrimp’s hemocoel by the recognition of diversified PAMPs located on these immunostimulants. Studies have investigated whether WSSV could be prevented by activating the immune system of the host [96,235]. This was done by administering immunostimulants derived from different sources as reviewed by Kumar et al. [235]: plants, bacteria, algae, fungi, animals, synthetic sources, nutritional factors, and hormones. Beta-glucans, peptidoglycans, or lipopolysaccharides are among the main proposed immunostimulants [234,294,304,305,306,307,308,309].

The methods used to supply immunostimulants can be direct or indirect. Direct methods are invasive, usually through subcutaneous, or intramuscular injection. Indirect delivery methods include immersion, using a cannula, or inclusion in the feed [277,306,310,311]. All methods mentioned, except for the latter, have the disadvantage of requiring the direct handling of the organisms, and are thus less attractive for their use by the industry [306]. The development of efficient oral immunostimulant delivery methods is therefore a necessity.

While administration of beta-glucan prior to WSSV infection has been shown to result in a reduction of mortality compared to the controls that did not receive beta-glucan [304,305,306,309,310,312], some studies also found that an overdose of beta-glucans led to high mortality rates in shrimp possibly caused by immunosuppression, immune fatigue, or by excess generation of reactive oxygen species (ROS) [96,278,304,305,311]. These studies concluded that the periodicity and dose to which the immunostimulants are supplied are two important factors determining these molecules’ protective effect [278,304,305,306]. Hence, further optimization of these factors using *in vivo* infection models is a must.

To test the efficacy of immunostimulants against WSSV infection, a natural inoculation method (immersion, feeding of WSSV-infected tissues) in combination with the individual housing of the experimental animals is put forward as the best option when there is a need to investigate the immune responses that are triggered (Table 4). Both the immunostimulants and WSSV are expected to provoke an immune reaction [235]. Indeed, several studies have, for instance, shown that TLRs (Toll-like receptors) were upregulated during WSSV infections in *P. monodon* (*PmToll*) [313,314], *M. japonicus* (*MjToll*) [315], *M. rosenbergii* (*MrToll*) [316], and *L. vannamei* (*LvToll*) [246,317,318]. The Toll pathway is one of the major signaling pathways that participates in the immune defense against WSSV infections in shrimp [299] and there are more signaling pathways such as the JAK/STAT that are reportedly also involved [80,319,320,321,322,323,324]. Using an inoculation method that mimics natural WSSV infection is therefore preferred, because specific tissue-resident hemocytes or immune responses might not be triggered if the natural defense barriers are breached by a more artificial inoculation method, as discussed in Section 2.3 [325]. Additionally, an *in vivo* experiment aimed at the assessment of the immune responses should always include at least one experimental treatment group that only receives the immunostimulant, a group that is only challenged with the virus, a group that is challenged with WSSV while receiving the immunostimulant treatment, and a control group that is not treated or challenged with anything (blank), so that the immune responses that are triggered can be assessed together and separately to differentiate them. This will lead to a more meaningful interpretation of the results. The evaluation of the immunostimulant’s protective properties against WSSV can be based on clinical observations such as the survival rates and pathogen counts [233,326,327], and this can in theory be done in a group challenge model. However, phenomenal observations *in vivo* are generally not sufficient to elucidate the protective immune responses that are triggered by the immunostimulant. It is therefore recommended to include additional immune parameters that can be evaluated through the model and/or additional laboratory tests. That is why individual housing of the animals is preferred over group housing, as it allows for the sampling of individual shrimp at different timepoints to test an array of different immune parameters. Immune parameters that can be assessed in an *in vivo* challenge model in shrimp include the total hemocyte count (THC), differential hemocyte count (DHC), phagocytic index (PI), immune enzymes specific activity (phenoloxidase activity (POA), superoxide dismutase activity (SOD), Lysozyme activity (LZA), intracellular respiratory burst activity (IRB)), and the expression of known immune-related genes such as *Hsp70*, *TGase*, *LYS*, and *ProPO* [292,328,329,330,331,332,333,334,335,336,337,338]. Once an immunostimulant has been tested in individual animals and efficacy has been established, it is advisable that the investigators also evaluate it in a group challenge model to further optimize the treatment under these conditions (refer to the sections Biologically Active Compounds and Antibody Therapy) (Table 4).

##### RNAi-Based Therapy

In shrimp, RNA interference (RNAi) is a highly conserved intracellular immune response triggered by the presence of double-stranded RNA (dsRNA). It leads to the silencing of genes post transcription to defend cells against parasitic nucleotide sequences from viruses or transposons [339,340,341]. RNAi is initiated by the double-stranded RNA (dsRNA)-specific endonuclease or Dicer, which promotes the cleavage of long dsRNA into 21–23-mer short interfering RNA (siRNA). Then, these siRNA molecules induce a sequence-specific degradation of homologous single-stranded (viral) mRNA, thereby representing one of the only known specific antiviral mechanisms in shrimp [230,233,342,343,344,345]. RNAi offers a targeted approach to interfere with the replication of infectious agents, such as WSSV, making it an attractive strategy for white spot disease (WSD) control in shrimp aquaculture [247,250,346,347,348,349,350,351,352,353,354]. For instance, *vp28-siRNA*s injected along with WSSV in *M. japonicus* were shown to inhibit WSSV replication [96,353,354]. Moreover, the use of *vp28* and *vp37*-dsRNA effectively eliminated WSSV virions from *M. japonicus* [355] and *L. vannamei* [179,250] challenged with WSSV [352]. Most studies have applied intramuscular RNAi administration [179,250,356,357,358,359,360,361,362,363,364,365]. However, this is not viable for applications on a large scale in shrimp farms [179]. The naked RNA degrades quickly when supplied in feed [357,358], either due to feed processing or the digestion process [359]. The challenge is to develop a treatment through the oral route [360,361] instead of through injection, yet one in which the RNA is nonetheless protected [179]. Moreover, when shrimp eat, they have the peculiar tendency to fragment feed pellets (due to their size and to food selectivity for palatability and hardness). This differs from fish, who swallow whole pellets. Therefore, a considerable amount RNA can be lost in the water while shrimp are feeding [364]. This suggests that a higher amount of RNA is needed for oral delivery than for intramuscular delivery to reach the same level of protection. So far, effective oral delivery of RNAi molecules has been achieved by a few strategies, including the intake of virus-like nanocarriers containing dsRNA [179], dsRNA-enriched bacteria [361,362], transgenic microalgae expressing dsRNA [363], and viable brine shrimp (*Artemia*) zygotes [352,364].

RNAi-based therapies specifically target the replication cycle of the virus. In other words, they aim to trigger the defense mechanism of RNAi to prevent virus multiplication in a host cell by supplying dsRNAs or siRNAs that encode WSSV-specific targets. They do not attempt to neutralize free virions like some of the treatment strategies that were previously discussed. It is therefore recommended to test the efficacy of these therapies directly *in vivo*, in the absence of immortal cell cultures. Individual housing of the experimental animals is advised in the early stages of delivery method and efficacy testing and formulation and dosage optimization, while the animals should be housed in group in the later stage of therapy development, as discussed in the sections Biologically Active Compounds and Antibody Therapy (Table 4). WSSV inoculation procedures that emulate natural infections in the field are preferred, since specific immune responses might not be triggered by a more artificial inoculation method, as mentioned previously [325] (Table 4).

##### Vaccination

Traditionally, immune mechanisms have been divided into two categories, innate and adaptive [365]. Vertebrates have both an innate system of defense and an acquired response, while extensive homology between vertebrates and invertebrates has only been found for the innate defense system [366,367,368]. Extensive searches in invertebrate taxa for B cells, T cells, and major histocompatibility complex molecules, the key ingredients of vertebrate acquired immunity, have not been successful [327]. This led to the dogma that invertebrates, including shrimp, lack immunological memory or specific immune responses and can therefore not be vaccinated [298,327,369,370]. However, several studies of immune defense in invertebrates demonstrated phenomena that are functionally equivalent to those in vertebrates, showing both immunity that is acquired and tremendous variation in the expression of disease that can be described as specificity [327]. To distinguish these phenomena from the classical immune memory triggered by T/B lymphocytes in vertebrates, the terms “immune priming”, “innate memory”, “learned immunity” or “trained immunity” are used to describe the immunological memory that arises from the innate immune responses after infection or vaccination [233,371,372]. Possible mechanisms behind trained immunity in shrimp include Dscam alternative splicing, and epigenetic modifications through DNA/RNA methylation, histone tail modifications, non-coding RNA (ncRNA), RNA interference (RNAi) or other mechanisms [373,374,375,376], followed by the inheritance of the acquired phenotypes by subsequent generations [377,378] as reviewed by Roy et al. [298]. Many *in vivo* “vaccination” experiments have now explicitly shown that primary exposure to pathogens may be prophylactic, providing hosts with protection during secondary encounters [234,310,379,380,381,382,383,384,385,386], and that these responses can be specific to pathogen genotypes. Moreover, these specific responses were sustained to outlast the general innate immune response, which implies the formation of an “immune memory” [117,387,388,389,390,391]. Hence, much interest has been focused on the memory-like immune responses of the innate immune system after vaccine-like treatment [233]. The term “vaccination” has been widely accepted and used in the literature on shrimp immunity [254,255]. It is defined by the stimulation of the immune response by antigens but should be distinguished from immunostimulation by substances such as LPS and β-glucan, because the protection they provide depends on general innate immunity, as opposed to antigenic immune specificity [233]. As reviewed by [233], vaccination against WSSV with live vaccines [386], inactivated virus vaccines [382,383,385,392], virus subunit vaccines [107,385,391,393], and DNA vaccines [394,395,396,397] has shown to lead to an increased protection post WSSV challenge in multiple studies lasting between 7 and 30 days [233]. To date, “vaccination” on the laboratory scale has been reported in almost all commercially farmed penaeid species. In contrast, field trials examining the efficacy of this approach in shrimp disease mitigation are almost non-existent [398]. This has been attributed to the fact that development of a suitable delivery strategies for the mass vaccination of shrimp in aquatic systems is a major challenge [399]. Oral administration to shrimp (i.e., through feed) is by far the most appealing method of vaccine delivery. Thus, new generation technologies are attempting to provide more efficient oral delivery systems, for instance by using baculovirus [111,400] or *Bacillus subtilis* spores [401,402], both modified to express VP28, as vehicles [399]. Proper evaluation of these new vaccine delivery systems under controlled laboratory conditions is a crucial first step towards field validation. Subsequently, vaccination can become one of the viable options for the management of WSSV infections in ponds and can be employed either singly, or in tandem with other approaches such as nutritional enhancement, immunostimulation, and application of biosecurity protocols [398].

A typical experiment to study vaccination against WSSV in shrimp starts with immune priming, e.g., an oral or intramuscular “vaccination” with live or inactivated virus, or with a subunit or DNA vaccine encoding structural proteins of WSSV. This priming is followed by a time delay and then a pathogen challenge with WSSV [111,383,384,386,395,396,403,404]. Another possibility is that an experiment includes immune priming, a time delay, one or more immune boosters, another time delay, and finally a WSSV challenge [107,382,385,390,391,393,397]. It is strongly recommended to use an inoculation method that mimics natural WSSV infection because, as mentioned in the section Nonspecific Immunomodulators, there is a chance that specific tissue-resident immune reactions might not be triggered by a more artificial inoculation method [325] (Table 4). Shrimp in vaccination experiments can be housed in groups if the aim is to mimic the realistic circumstances of a WSSV outbreak to evaluate the protective effect of candidate vaccines on a population of shrimp (Table 4). However, if an investigator wished to research the fundamental mechanisms behind trained immunity induced by vaccines against WSSV, it is preferable to opt for individual housing (Table 4). This provides a more controllable experimental setting that rules out variables caused by cannibalism and secondary host transmission [391], and it allows for the sampling of individual shrimp at different timepoints as discussed previously in the section Nonspecific Immunomodulators.

#### 3.4.3. Genetic Selection

Although the development of antivirals and immunomodulators holds promise for the prevention and treatment of WSD, to the best of our knowledge, the effectiveness of these control measures has not yet been clearly proven under field conditions. An alternative approach that has been proposed is to improve WSD-resistance in shrimp species through selective breeding [210,211,213] (refer to Section 3.1.2). Indeed, survival during an epidemic is partly determined by host traits [405], namely (i) disease resistance (an individual’s propensity to avoid becoming infected or diseased), (ii) endurance (the propensity of diseased individual to survive the infection), and (iii) infectivity (i.e., the propensity of an infected individual to transmit disease) [405]. For the estimation of genetic parameters of WSSV resistance and endurance in shrimp, the use of infection models in which shrimp are housed in groups and challenged with WSSV-infected tissues has been critiqued. Gitterle et al. [113] stated that the animals in this type of infection model are not exposed to the same risk of infection, since it is highly unlikely that all animals will eat the same amount of tissue. Additionally, the time period between risk of infection and the event of death is difficult to determine because the animals are also not likely to eat the tissues at precisely the same time. Therefore, it can be complicated to make a correlation with the innate genetic ability of some animals to resist or ameliorate infection by the virus [113]. It is therefore recommended to opt for a challenge model in which all the test animals could be assumed to be equally at risk of being infected at the same time (because both the binary mortality data and the days of survival can be used for the analysis). Gitterle et al. [113] proposed that a protocol that uses immersion or oral intubation (refer to Section 2.3) to infect host populations would be suitable. However, they did not consider that housing the animals in groups would still facilitate transmission between the animals, thereby exposing some animals to a higher risk than others [102,113]. To ensure that the infection in individuals is controlled, a combination of individual housing and individual inoculation (immersion, oral intubation, and individual feeding of WSSV-infected tissues) is recommended (Table 5). Another benefit of housing the shrimp individually, is that the animals can be easily sampled, as mentioned in the section Nonspecific Immunomodulators. However, although this might be the best approach to estimate genetic parameters of resistance and endurance in individual shrimp, it might not be the most optimal method to select for low infectivity [213]. The ultimate goal of selective breeding for disease traits is to reduce the risk of an epidemic [406]. In epidemiology, the key parameter determining the risk and size of an epidemic is the basic reproduction ratio, R_0_. This is the average number of secondary cases produced by a typical infectious individual during its entire infectious lifetime, in an otherwise naïve population [407]. R_0_ has a threshold value of 1. When R_0_ < 1, the epidemic will die out. On the other hand, when R_0_ > 1, major outbreaks can occur [102,406]. Hence, breeding strategies to reduce the risk and prevalence of an infectious disease should aim at reducing R_0_, preferably to below a value of 1 [406]. While this is probably the obvious choice for epidemiologists, it may be unexpected for breeders who are not very familiar with R_0_ [406]. Breeding to reduce R_0_ raises a conceptual difference between quantitative genetics and epidemiology: R_0_ is an epidemiological parameter referring to an entire population, whereas quantitative genetics rests on the concept of individual breeding value (i.e., the deviation between the mean value of an individual’s progeny and the mean value of a reference population for a particular trait) [406,408,409,410]. Nevertheless, R_0_ as an epidemiological parameter is strongly affected by the variation in susceptibility and infectivity between hosts and this variation is influenced by differences in individual genotypes [409]. Hence, in a genetically heterogeneous population, R_0_ is a function of individual genotypes in the population, which in turn are a function of allele frequencies. Moreover, a change in allele frequencies will change R_0_, indicating R_0_ can respond to selection [406]. Genetic improvement aiming to reduce R_0_ should ideally be based on the effects of an individual’s genes on R_0_. This would require defining the individual breeding values for WSSV susceptibility and infectivity. Moreover, defining these breeding values would also allow defining heritable variation in WSSV susceptibility and infectivity, that is, the variation in individual breeding values for WSSV susceptibility and infectivity, which measures the potential for response in those traits and consequently also the response in R_0_ [406]. Estimation of genetic parameters for WSD resistance/susceptibility can be conducted in an individual WSSV challenge model, perhaps to subsequently estimate the degree of genomic relationship between test animals and individual candidate breeding animals within families to estimate their individual genomic breeding values [213]. After all, the test animals themselves are rarely incorporated in breeding programs, because they might vertically transmit the virus to their offspring [213,226]. Selection for host infectivity, on the other hand, requires that animals are maintained in groups in an environment that encourages reproduction of WSD, and individual inoculation is of lesser importance [213] (Table 5). Moreover, infectivity and susceptibility exhibit indirect genetic effects (IGEs), these are heritable effects of an individual on the trait value of another individual. Indeed, an individual’s susceptibility and infectiveness affect the disease status of its contacts [406,411,412,413,414,415]. Research in the field of IGEs suggests that group selection and relatedness among interacting individuals (“kin selection”) can be used to increase the response to selection for a reduction in R_0_ in two ways. First, if, by chance, a test animal carries an allele for low infectivity in a group of genetically related conspecifics, the chance is high that the infectivity of these group mates is also below average. This increases the individual’s probability of escaping the epidemic, and thus being selected for breeding. Second, if group mates are related, an individual that carries an allele for low susceptibility has on average also fewer infected group mates, which increases its probability of escaping the epidemic and being selected. The net result of both mechanisms is a strong increase in response to selection in R_0_ when relatedness increases [406,411,416]. Hence, it is recommended to carry out genetic selection studies for a favorable R_0_ in populations that are housed in groups in such a way that group mates show a certain degree of genetic similarity, because this is expected to yield a substantially greater response (Table 5). The use of an inoculation procedure that mimics natural transmission is strongly preferred (refer to Section 2.3 and Section 3.1.2). Families with lower WSSV susceptibility and infectivity would be expected to show slower transmission rates and flatter survival curves compared to families with less favorable genes for these traits.

#### 3.4.4. Artificial Intelligence

Recently, researchers are attempting to leverage the benefits of AI in various ways to control WSD. Liu et al. [240] for instance, suggest that the innovative fusion of predictive modeling and smart nanotechnology, offers a cutting-edge approach to combat WSD, because it enables precise drug delivery and targeted interventions at the molecular level. Moreover, some researchers are developing machine learning models for the prediction of the occurrence of disease, because based on such information, shrimp farmers could easily determine suitable locations for new farms or prepare appropriate solutions to avoid infection [239,243]. Lastly, machine learning methods to enhance early WSD detection in shrimp using computer vision systems and image analysis algorithms have been proposed, although limited visibility combined with bottom dwelling behavior may present unique challenges [236,237,241,242]. Biological WSSV infection models could be valuable tools to train WSSV recognition models. This may involve monitoring WSSV challenge experiments, capturing images at regular intervals to identify early signs of WSSV, and exploring intervention efficacy [236]. Such experiments could provide valuable insights, enhancing the accuracy and reliability of the WSSV recognition model for more precise and timely disease detection [236].

## 4. Concluding Remarks

From this review, it should be evident that *in vivo* infection models are indispensable and powerful tools for the advancement of WSSV research [51,53,88,102,120], especially since continuous cell lines from marine invertebrates in general [417], and shrimp in particular [13], are not available yet. Animal models have been used to determine the pathogenicity of WSSV, host susceptibility, to study aspects of the pathogenesis, transmission, and epidemiology, and to investigate host immune reactions and the efficacy of anti-WSSV products [120]. However, all aspects of the model should be well thought-out to use it with confidence and different infection models might be considered for different lines of WSSV research.

## Figures and Tables

**Table 1:** Factors of shrimp pathogen challenge experiments that require standardization and transparent reporting (adapted from Arbon et al. [56]).

Factor	Experimental Detail
Host	
Age	Stage, including days post hatch (dph) or days of culture (DOC)
Size	Weight (g) and/or length (mm)
Source	Habitat (farmed or wild, hatchery, pond, etc.) Genetic/geographic/temporal source (country, region, date collected)
Acclimation	Duration (days) Conditions
Pathogen pre-screening	Sample size screened (proportion of experimental cohort) Pathogens screened Method of screening (PCR, qPCR, etc.) Screening results (pathogen load and prevalence)
Pathogen	
Source (strain)	Genetic/geographic/temporal source (country, region, date collected) Genetic sequence and strain identification (if available)
Processing method	Tissue or hemolymph sampling Homogenization, filtration, clarification, purification, etc.
Pathogen pre-screened	Pathogens screened Method of screening (PCR, qPCR, etc.) Screening results (pathogen load and prevalence)
Volume and concentration	Volume of inoculum used Viral copies per unit volume
Inoculation method and conditions	Co-habitation: exposure duration, removal of mortalities, holding configuration Immersion: bath duration and concentration, washing post bath Intrabladder: device and procedure Intramuscular injection: injection site Peroral intubation: device and procedure Feeding on infected tissue: starvation period, feeding period, volume of tissue (%BW)
Control inoculum	Control treatment used If nonstandard control is used: pathogens screened, method of screening, screening results (pathogen load)
Environment	
Experimental design	Definition of experimental and control groups Duration of the experiment (hours post infection—hpi)
Replication	Tank size Stocking density (as the number of shrimp per unit of area or as the liveweight per unit of area) Tanks per treatment Treatments within the experiment Repetition of the experiment
Sampling and analysis	Sample tissue type Sampling technique Sample storage conditions Sampling schedule (hpi) Shrimp replacement (during sampling and for mortalities/moribund shrimp) Details of laboratory and statistical analysis performed
Environmental conditions/water quality	Temperature Salinity Alkalinity pH DO Nitrogenous compounds (e.g., TAN-N, NO_3_, NO_2_) Aeration provisions Filtration and water treatment provisions
Feeding	Feed source and pathogen screening results Feed treatment provisions Feeding schedule (BW% per feeding event)

**Table 2:** Established WSSV inoculation methods, their advantages, and limitations.

Inoculation Method	Pro	Contra	Shrimp Species	Reference(s)
Co-habitation with sick shrimp in the absence of cannibalism	- Water-borne transmission can occur, while transmission through ingestion of infected tissues is prevented. - Allows study of water-borne transmission dynamics.	- Efficiency is low. - Difficult to prevent cannibalism unless the shrimp are housed in a specialized set-up that separates the individual animals but allows passage of WSSV-contaminated water.	*L. setiferus*	[97]
			*L. vannamei*	[97,101]
			*P. monodon*	[101]
Immersion in liquid viral stock	- Mimics natural water-borne transmission. - More controlled than co-habitation with sick shrimp.	- Efficiency varies between studies. - Experiment set-up can be challenging considering the desired titer and the volume of viral stock that needs to be produced to reach it.	*L. vannamei*	[51,95,103,104,105]
			*Macrobrachium. rosenbergii*	[106]
			*Marsupenaeus japonicus*	[1,89,107,108]
			*P. indicus*	[109]
			*P. monodon*	[110,111,112]
Immersion in infected rearing water	- Emulates natural water-borne transmission. - Effective in the studies that have used it.	- Efficiency was reportedly high. - Not practical, because the infected rearing water from a tank/aquarium in which WSSV-infected animals were housed must be used to inoculate naïve animals.	*L. vannamei*	[102,113]
Intrabladder	- Very efficient. - Precise administration of fixed quantity of virus. - Administration via one of the reported natural infection routes.	- Requires more experience than other methods. - Requires specialized devices, otherwise there is a higher risk of perforation. - Potentially difficult to perform in smaller shrimp.	*L. vannamei*	[10,11,99]
Intramuscular injection	- Most efficient method to infect shrimp. - Precise administration of fixed quantity of virus. - It requires some experience, but it might be easier to perform than peroral intubation or intrabladder inoculation, because perforation is the goal of this method, and the use of smaller sized shrimp is less problematic.	- Because it by-passes the shrimp’s natural defenses, this method might not represent one of the natural transmission routes.	*L. vannamei*	[42,51,53,76,95,105,114,115]
			*M. rosenbergii*	[106,116]
			*M. japonicus*	[107]
			*P. monodon*	[117,118]
			*P. semisulcatus*	[119]
Peroral intubation	- Precise administration of fixed quantity of virus. - Administration via one of the reported natural infection routes.	- Efficiency is reportedly low. - Requires experience since it can be a challenging procedure to perform. - Requires specialized devices, otherwise there is a risk of perforation. - Risk of regurgitation of the inoculum, which invalidates the benefit. - Potentially difficult to perform in smaller shrimp.	*L. vannamei*	[76,98,120]
Feeding on infected tissue	- Mimics natural WSSV transmission.	- Efficiency varies between studies. - Risk that the inoculum is not homogeneous. - Not every shrimp consumes the same amount of inoculum at the same time. - Comparison between studies can be complicated by variations in inoculum production procedures.	*L. setiferus*	[97]
			*L. vannamei*	[76,88,102,109,121]
			*M. rosenbergii*	[106]
			*M. japonicus*	[107]
			*P. indicus*	[109]
			*P. monodon*	[109,122]

**Table 3:** Recommended *in vivo* research methods for antiviral therapies against WSSV in shrimp.

Control Measure	Examples	Basic Mechanism(s) of Action	Research Need(s)	Recommended *In Vivo* Research Methods
Antiviral Therapy	- Biologically active compound. - Antibody therapy (passive vaccination).	- Viral inactivation due to interaction with the viral envelope proteins. - Interference with steps in the replication cycle of the virus, which prevents WSSV multiplication in the host cells.	1. Capacity to neutralize free WSSV virions *in vitro.*	Antiviral activity test: *in vitro* incubation of the mixture (virus + antiviral agent) followed by *in vivo* titration in individually housed shrimp (refer to Section 3.1.1).
			2. Protective activity *in vivo.*	Challenging individually housed shrimp (refer to Section 2.4) with an inoculation procedure that mimics natural transmission (refer to Section 2.3) and administering the antiviral agent before or after the challenge depending on the proposed mechanism of action.
			3. Efficacy of group treatment.	Challenging shrimp housed in a group (refer to Section 2.4) with an inoculation procedure that mimics natural transmission (refer to Section 2.3) and administering the antiviral agent before or after the challenge depending on the proposed mechanism of action.

**Table 4:** Recommended *in vivo* research methods for immunomodulators against WSSV in shrimp.

Control Measure	Examples	Basic Mechanism(s) of Action	Research Need(s)	Recommended *In Vivo* Research Methods
Immunomodulators	- Nonspecific immunostimulants. - RNAi-based therapy. - Vaccination.	- Stimulating the host immune response without directing the activity of stimulated cells to a specific antigen. - Stimulating the host immune reaction to a specific antigen.	1. Protective capacity *in vivo* due to immunomodulatory activity.	Challenging individually housed shrimp (refer to Section 2.4) with an inoculation procedure that mimics natural transmission (refer to Section 2.3) and administering the immunomodulator before or after the challenge depending on the proposed mechanism of action.
			2. Efficacy of group treatment.	Challenging shrimp housed in a group (refer to Section 2.4) with an inoculation procedure that mimics natural transmission (refer to Section 2.3) and administering the antiviral agent before or after the challenge depending on the proposed mechanism of action.

**Table 5:** Recommended *in vivo* research methods for selection of WSSV-resistant shrimp.

Control Measure	Research Need(s)	Recommended *In Vivo* Research Methods
Genetic selection for WSSV resistance	1. Selection for disease resistance and endurance.	Challenging genetically diverse, individually housed shrimp with an inoculation procedure that mimics natural transmission, but that allows the shrimp to be equally at risk of being infected and at the same time (refer to Section 2.3 and Section 3.1.2).
	2. Selection for a reduction in the basic reproduction number, R_0_.	Challenging genetically related shrimp housed in groups (refer to Section 2.4) with an inoculation procedure that mimics natural transmission (refer to Section 2.3 and Section 3.1.2).
